# A Candidate Verification Gate for Reducing False-Positive Staging in a Pressure Ulcer AI Pipeline

**DOI:** 10.3390/diagnostics16203324

**Published:** 2026-10-13

**Authors:** Dong-Hun Han, Jun-Woo Choi, Kyu Hyun Nam, Minsoo Kang

**Affiliations:** 1 Department of Medical Artificial Intelligence, Eulji University, Seongnam 13135, Republic of Korea; d555v@naver.com (D.-H.H.); chlwnsdn0214@gmail.com (J.-W.C.); nanmider44@gmail.com (K.H.N.); 2 MDIS Co., Ltd., Seongnam 13135, Republic of Korea; 3 School of Advanced Eng., Eulji University, Seongnam 13135, Republic of Korea

**Keywords:** pressure ulcer, candidate verification gate, false-positive suppression, hard-negative mining, retrospective challenge set, source-image separation, sensitivity tradeoff

## Abstract

**Background/Objectives:** A four-class stage classifier can assign a pressure ulcer stage to an erroneous detection. We evaluated supervised candidate verification and its rejection costs. **Methods:** This retrospective challenge-set study used a frozen detector and stage classifier with an existing binary gate threshold of 0.70. Negative evaluation data comprised 1138 Normal-labeled skin images from 347 source photographs and 328 non-pressure-wound images. A stronger source-family audit reduced the original 112-image positive subset to 94 images from 93 families; these still overlapped detector development. Additional experiments compared three source-grouped binary classifiers and paired detector fine-tuning with or without mined negative images. **Results:** The historical gate reduced skin images with false-positive outputs from 269/1138 (23.6%) to 54/1138 (4.7%). In the corrected positive subset, 106/107 detected lesions were retained; overall lesion recall changed from 107/110 to 106/110. Both historical false-negative lesions remain in the safety review. Newly trained You Only Look Once (YOLO) 26s, ResNet18, and MobileNetV3-small models achieved internal test-positive retention of 97.4–98.5% after separate calibration. Adding mined negatives directly to detector fine-tuning also reduced false-positive outputs. The historical gate accepted 122/147 matched non-pressure-wound candidates. **Conclusions:** Negative supervision reduced false-positive outputs through both modular verification and detector retraining. The findings do not establish superiority of a separate gate, patient-level generalization, clinical acceptability of missed lesions, or pressure ulcer etiology discrimination.

## 1. Introduction

Pressure ulcers, also termed pressure injuries, are localized injuries to skin or underlying tissue associated with pressure or pressure combined with shear. A meta-analysis estimated a 12.8% pooled prevalence among hospitalized adults [1]. The National Pressure Injury Advisory Panel (NPIAP), European Pressure Ulcer Advisory Panel (EPUAP), and Pan Pacific Pressure Injury Alliance distinguish Stages 1–4, unstageable pressure injury, and deep tissue pressure injury (DTPI) [2,3]. Staging supports assessment and management after pressure etiology has been established; it is not an etiologic diagnosis. Variability in visual assessment [4,5] motivates image-based assistance, but an erroneous stage on normal skin or a different wound can also misdirect interpretation.

Pressure ulcer image models have been evaluated with convolutional classifiers, object detectors, and smartphone applications [5,6,7,8,9]. Their strengths include automated localization and access at the point of care; their reported accuracy is nevertheless conditional on the case mix, annotation quality, and separation of development and evaluation data. Reviews of wound imaging [10] and work on diabetic foot ulcer detection [11,12] also show why wound localization and wound etiology are distinct tasks. Our earlier studies addressed staging generalization and clinical deployment [13,14]. Those results do not transfer to the present verifier, whose training, failure modes, and evaluation endpoints differ.

### Related Work and Study Scope

The literature already includes explicit non-pressure ulcer handling. Aldughayfiq et al. [6], for example, used five classes, including a non-pressure ulcer category. Consequently, neither binary lesion screening nor adding a background class is novel in itself. However, a background-aware detector or classifier and a separately inserted verifier represent different implementation choices. In an existing four-stage pipeline, a false detection is necessarily assigned one of the available stages unless a rejection decision intervenes. We define false-positive staging at first use as any Stage 1–4 output on an evaluation image or candidate labeled as not containing a pressure ulcer; this is an output error, not a measured treatment decision.

Reject-option classification balances coverage against error and rejection costs [15,16]. Open-set recognition [17,18] and out-of-distribution scoring with maximum softmax probability (MSP), temperature scaling with input perturbation, feature distance, or energy [19,20,21,22] offer related approaches. These methods differ in their information and training requirements: a confidence score from an existing classifier has not necessarily learned the particular background errors made by a deployed detector. Conversely, a binary classifier trained on those errors benefits from labeled negatives and should not be presented as an intrinsically superior open-set algorithm. Experimental work shows that the quality of the underlying classifier materially affects open-set results [23], while a broader survey describes the variety of detection settings and assumptions [24]. Medical dataset shift further limits transfer between sources [25].

Supervised negative exposure provides another relevant comparison. Outlier exposure trains with auxiliary atypical inputs [26], whereas the present gate learns the accept/reject labels of detector-related crops. Source effects also require explicit evaluation: cross-institution radiograph studies [27] and skin-image instrument-bias experiments [28] show that useful-looking discrimination can depend on acquisition cues. These are methodological precedents from other settings, not evidence that the present gate has or has not learned such shortcuts.

The specific question was whether adding a supervised candidate verification gate to a frozen detector and stage classifier reduces false-positive outputs on held-out negative images, and what proportion of previously detected true lesions it removes. We examined whether detector-derived negative crops provide rejection information beyond existing confidence scores. These are retrospective questions, not a prospectively registered clinical hypothesis. The contribution is an application-level analysis of a modular intervention, including overlap correction, separately calibrated supervised baselines, detector retraining, and failure review. The gate is a conventional binary classifier; no new architecture, learning objective, or open-set recognition algorithm is claimed.

The detector and stage classifier are treated as previously developed components of the deployed application. Their study-specific training and export settings are documented here for reproducibility, but their weights were frozen for the original gate experiment. The candidate verification gate is the component introduced to address the failure examined in this manuscript. The 1466 negative evaluation images were not used to fit these models or choose the existing threshold. The positive images, in contrast, originate from the detector development data. This asymmetry limits the question to retrospective false-positive suppression and associated retention, rather than independent validation of the entire staging pipeline.

## 2. Materials and Methods

### 2.1. Target System and Ethics Approval

The target was the inference pipeline of an Android application deployed at Eulji University Hospital in Daejeon. The study was conducted in accordance with the Declaration of Helsinki and approved by the Institutional Review Board (IRB) of Eulji University Hospital in Daejeon (approval no. EMC IRB 2025-06-003, approved on 19 June 2025). The IRB waived informed consent for the retrospective analysis of de-identified data without intervention or direct patient contact. We use the Checklist for Artificial Intelligence in Medical Imaging (CLAIM) to organize reporting [29,30]. There was no prospective clinical evaluation of the gate. Reference annotations were inherited rather than independently adjudicated for this study, which is a limitation rather than an exemption from reference-standard assessment.

### 2.2. Proposed Pipeline

**Figure 1:**
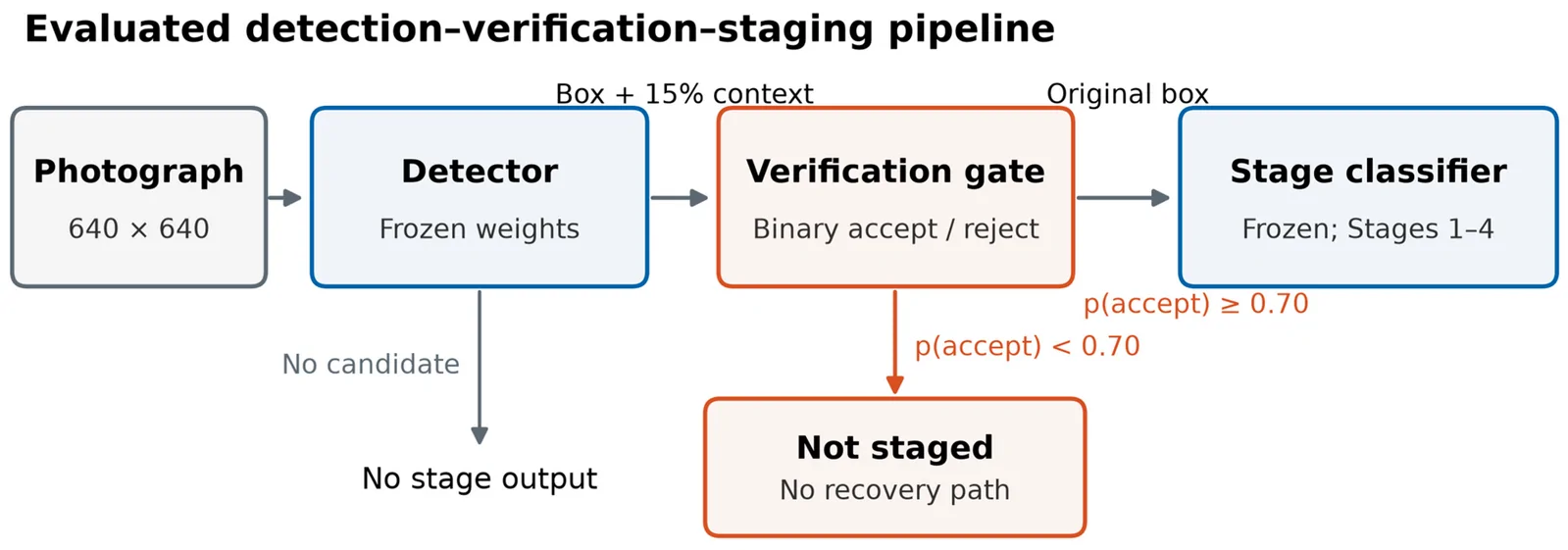
Candidate verification within the frozen detection and staging pipeline. Only accepted candidates reach the stage classifier. Rejected candidates are not staged and may include true lesions. The Figure depicts the evaluated automated path; a clinician review or recovery mechanism was not implemented in this experiment.

The evaluated cascade comprises detection, verification, and staging (Figure 1). The image is letterboxed to 640 × 640 pixels. Candidate boxes above detection confidence 0.20 undergo class-agnostic duplicate removal at intersection over union (IoU) 0.6. A crop expanded by a total context margin of 15% is processed at 224 × 224 pixels by the candidate verification gate. A candidate with an acceptance score of at least 0.70 proceeds to the frozen stage classifier using the unexpanded box. A rejected candidate receives no stage. Rejection can therefore remove a true lesion and must not be interpreted as confirmation that a lesion is absent.

The gate labels define an operational training task: accept denotes a lesion crop and reject denotes a sampled background crop, including detector-derived false detections. Its score is denoted P (accept). It is not a calibrated probability of pressure ulcer etiology, and acceptance does not establish that NPIAP/EPUAP staging is appropriate.

The gate decision is defined in Equation (1). Here, *g*_accept_ is the acceptance probability produced by the gate, *c* is a crop with the context margin, and *τ*_g_ is the pass threshold:

*s*(*c*) = *g*_accept_(*c*), accept(*c*) = **1**[ *s*(*c*) ≥ *τ*_g_ ] (1)

The three deployed components use Ultralytics [31] You Only Look Once (YOLO) 26-family architectures [32]. Model-specific initialization, data roles, and training settings are reported separately to distinguish prior component development from the new verification experiment (Table 1 and Table 2). Although the detector has a four-stage head, the evaluated pipeline uses its coordinates and confidence; the stage classifier supplies the displayed stage. Study-specific fine-tuning must not be confused with training from random initialization.

### 2.3. Construction of Training Data for the Verification Gate

Training the gate required many lesion-free images, which were not sufficiently available in the institutional data. We therefore collected 1545 images from the validation split of Open Images V7 [33] in categories likely to elicit false positives: seven lesion-free skin categories (human arm, leg, foot, hand, ear, nose, and face) and 20 categories of red or round objects (including pen, tomato, strawberry, apple, balloon, rose, traffic light, lipstick, and band-aid). Based on filename hashes, 1290 images were assigned to the mining pool, and 255 were reserved exclusively for measurement.

Hard negatives were defined operationally as candidate boxes produced by a fixed detector on the negative mining pool at confidence ≥ 0.05, using a total context margin of 0.15. This is offline detector-response mining, inspired by focusing on difficult examples [34], not online high-loss mining during detector training. The saved mining manifest records 889 of 1290 images with detections and 2940 mined crops. The dataset also contains 802 random crops from the remaining 401 images, two per image, so the 3742 rejected crops do not all come from the 889 detected images. Random crops use 25–65% of each image dimension. The original study reported one-investigator visual screening; no independent clinical adjudication or agreement statistic is available.

The available gate-development snapshot comprises 4262 accept and 3279 reject training crops, plus 375 accept and 463 reject validation crops. Reject training contains 2577 mined and 702 random crops; validation contains 363 mined and 100 random crops. A revision audit using source-content hashes found 1131 training and 159 validation negative sources with zero intersection. The saved builder assigns sources by hash before saving crops. This contradicts the original text describing crop-level reject splitting. Because a directory snapshot is not an immutable historical training manifest, we withdraw that explanation and the original 0.988 validation accuracy as confirmatory evidence. Section 2.11 gives a new fully recorded source-grouped experiment with separate calibration and test partitions. The available accept inventory contains 3730 public pressure ulcer crops from 563 normalized source families and 907 institutional crops from 704 families, totaling 4637 accept crops. These counts describe the saved gate inventory, not the larger stage-classifier dataset or certified immutable historical membership.

### 2.4. Separation of Cropping Protocols by Pipeline Stage

The gate receives a detection crop with surrounding context, whereas the stage classifier receives the unexpanded box. This tests the hypothesis that a lesion and its surroundings jointly inform candidate acceptance. It does not isolate whether the gate uses morphology-, background-, or acquisition-related cues. A crop with total margin ratio m is defined in Equation (2); Section 3.6 examines inference-time margins without retraining:

*c*_m_ = *I* [*y*_1_ − (*m*/2)*h*: *y*_2_ + (*m*/2)*h*, *x*_1_ − (*m*/2)*w*: *x*_2_ + (*m*/2)*w*] (2)

Here, (x1, y1, x2, y2) are the detection-box coordinates, and w and h are the box width and height. Coordinates extending beyond the image boundary are clipped. Because m denotes the total proportional increase along each axis, m = 0.15 adds 7.5% to each side. The evaluated gate uses m = 0.15 at inference. All 907 saved institutional accept crops have dimensions consistent with this same margin when compared with the original images and reference boxes; a total of 903 distinguish 0.15 from 0.08, and four boundary-clipped crops match both. Public accept crops were inherited as supplied. The submitted statement that institutional accept crops used m = 0.08 is therefore corrected; immutable historical crop-generation records remain unavailable. Section 3.6 varies the inference margin only.

### 2.5. Existing Operating Point and Exploratory Threshold Analysis

Equation (3) expresses the development objective of reducing false-positive outputs subject to retaining detected lesions. N denotes negative images, P denotes true-positive candidates, and Rmin is the empirical development retention requirement. This is a design objective, not a clinically validated utility function:

*τ*_g_* = arg min FP(*N*; *τ*_g_) subject to *R*(*P*; *τ*_g_) ≥ *R*_min_ (3)

The value τg = 0.70 was already configured in the deployed application before the present challenge-set analysis. The original development procedure sought 100% empirical retention on its internal benchmark while suppressing false positives. We therefore refer to 0.70 as the existing operational threshold. It was neither selected using an independent patient cohort nor justified by a prospective clinical noninferiority margin. It should not be described as universally optimal or clinically validated.

The historical benchmark included 255 negative images from Open Images and 199 true-positive candidates from detector development validation. Twenty-five of these candidates came from 32 images also present in the complete POS-PI pool. The originally reported 112-image subset was selected from detector-training images and did not enter that benchmark by exact image identity. The stronger source-family screen described below reduces this subset to 94 images; historical calibration membership cannot be certified at patient level. Thus, avoiding direct threshold-calibration reuse does not establish independence from upstream development. Original threshold sweeps remain exploratory; the new source-separated calibration experiment is described in Section 2.11.

Missing a pressure ulcer and displaying an unnecessary stage have different potential consequences, but their relative clinical costs were not measured. The constrained formulation records a development preference for retention. It does not establish that two additional missed lesions, or any particular recall decrease, are clinically acceptable.

### 2.6. Evaluation Data and Overlap Audit

The evaluation comprises negative skin images (NEG-SKIN), non-pressure wounds (WOUND-NPI), and pressure ulcers (POS-PI), summarized in Table 3 and Table 4. The overlap audit combined filenames with low-resolution image signatures. Signatures were generated from 24 × 24 RGB representations with channel and L2 normalization; the minimum cosine distance across horizontal and vertical flip combinations was screened at 0.07. This heuristic can detect some identical or related images, but cannot establish patient identity or guarantee that all near-duplicates were found. The audit identified 47 images in a 276-image detector-development candidate pool that matched WOUND-NPI. They had not been assigned to detector training or validation and were excluded from POS-PI, leaving 229 images.

NEG-SKIN comprises 1138 images labeled Normal in Ulcer Classification v2 (Roboflow Universe, LookInside workspace, release 8 April 2025, CC BY 4.0; https://universe.roboflow.com/lookinside/ulcer-classification/dataset/2 (accessed on 12 August 2026)). The images derive from 347 source photographs and include augmented copies with flips, rotations, and blur. WOUND-NPI comprises 328 images with 355 wound boxes from Foot Ulcer v2 (medica workspace, release 21 December 2024, MIT License; https://universe.roboflow.com/medica/foot-ulcer-10nf1/dataset/2 (accessed on 12 August 2026)). Both releases use 640 × 640 images. Their shared hosting platform and resolution do not establish shared clinical acquisition. The institutional positive images are not publicly available. Normal and non-pressure-wound designations are inherited dataset labels, not new clinical diagnoses.

No overlap was identified between the 1466 negative evaluation images and the training, validation, or threshold-calibration material used by the three deployed components under the available audit. These negative challenge-set estimates are not directly contaminated by the known positive-data overlap. However, filename and image-signature checks do not establish patient or institution independence, and the public datasets are not a representative prospective sample of clinical use.

All 229 positive evaluation images came from detector development: 197 from training and 32 from validation. Their 311 reference lesions also contributed stage-classifier development crops (271 training and 40 validation crops). Staging accuracy is therefore not estimated here. The gate development material included 134 crops from 117 of these images: 85 images contributed gate training and 32 gate validation. This provenance makes the full positive pool unsuitable for unbiased pipeline sensitivity or staging-performance estimation.

The original submission defined POS-PI-G as 112 images with 136 lesions excluded from gate development by exact identifiers. Revision auditing stripped repeated annotation-export prefixes and public augmentation suffixes to group related files. The 112 files represented 111 source families, and 18 files shared a normalized institutional source identifier with the available gate-development material. Conservatively excluding those files produced POS-PI-GS: 94 images, 93 source families, 110 reference lesions, and 107 detected true-positive candidates. The exclusion rule was based on provenance rather than gate outcome. Historical checkpoint membership cannot be fully reconstructed from a mutable directory snapshot, so this is a conservative correction against available artifacts, not a guarantee that all leakage was removed.

POS-PI-GS is the primary positive subset in the revised challenge analysis; POS-PI-G is retained only as a historical sensitivity analysis. All 94 corrected images remain in detector training and contributed to stage-classifier development crops. Candidate localization and selection may therefore benefit from prior exposure. Patient identifiers were unavailable, and source-family rules cannot identify different photographs of the same person or wound episode. Neither subset is an independent clinical test cohort.

### 2.7. Reference Standard and Annotation

For POS-PI images, a wound-care specialist nurse in the Quality Improvement Office of the institution established the stage according to NPIAP/EPUAP criteria, and the adjudicated stage label was encoded in the file metadata. A trained investigator annotated lesion bounding boxes using Label Studio, and the responsible clinician reviewed the annotations periodically. This procedure was the same as that established in our previous studies [13,14]. The images were not reannotated for the present study.

For revised stage-specific retention, detector-matched reference boxes were linked to the original per-lesion YOLO annotation classes by coordinate matching. All 311 reference boxes could be linked, with minimum matching IoU above 0.992. This corrects the original use of image-filename stage labels, which can misclassify individual lesions in images containing multiple annotated stages. This linkage is a provenance correction, not a new clinical reference-standard adjudication.

NEG-SKIN and WOUND-NPI retain the original public normal/ulcer labels and wound boxes. An emitted Stage 1–4 output on a correctly labeled negative image is counted as false-positive staging even when another skin finding is present. These labels were not independently adjudicated by clinicians for this study. Label error, uncertain wound etiology, and unavailable interobserver agreement can bias the estimated false-positive rate. The appearance taxonomy does not reassign clinical reference labels.

### 2.8. Inference Reproduction and Equivalence Verification

Inference reproduction used NumPy, OpenCV, Pillow, and Open Neural Network Exchange (ONNX) Runtime. Detector input uses letterboxing with gray padding 114 and bilinear downsampling. Gate and stage inputs resize the short side to 224 and center-crop to 224 × 224; repeated half-size reductions approximate antialiased downsampling. Pixel intensities are divided by 255. The end-to-end detector output is decoded without conventional nonmaximum suppression, followed by class-agnostic duplicate removal at IoU 0.6. Candidate matching and threshold comparisons use retained raw scores. Newly trained gate baselines use the explicitly documented torchvision transforms in Section 2.11 and are distinct from the historical application-equivalence test.

Training settings for the three models are provided in Table 2, and the crop-generation protocols are described with the corresponding models. Training and export used Ultralytics 8.4.96 and PyTorch 2.6.0. ONNX models were exported with opset 17, simplify = True, dynamic = False, a fixed batch size of 1, and no embedded nonmaximum suppression. The deployed application used ONNX Runtime for Android 1.20.0 without a registered hardware delegate.

Equivalence with the deployed system was verified using three reference images used in application instrumentation tests. The maximum box-coordinate discrepancy was 1 pixel, and the maximum discrepancies in stage confidence and detection confidence were 0.002, within the instrumentation-test tolerance of ±0.02. The gate also passed both positive reference images with an acceptance score of 1.000 and rejected the negative reference image with a score of 0.000.

### 2.9. Comparators

The reproducible score comparisons include MSP, negative predictive entropy (NENT), and approximate temperature-scaled MSP, alongside the detector-threshold sweep. Equation (4) defines these scores, with p denoting the normalized saved stage probabilities and T = 1000. Larger values favor acceptance. All methods are evaluated on the same corrected positive candidates and negative candidates for a given comparison. The gate alone was trained with negative labels; these comparisons therefore evaluate practical retrofit options, not matched-supervision architectural superiority:

sMSP = max p; sNENT = Σ p log p; sTS ≈ max softmax(log(max(p, 10^−12^))/T), T = 1000 (4)

The revision reconstructs temperature-scaled MSP from log(max(p, 10^−12^)) after normalizing the rounded stored probabilities. This is an approximation to temperature scaling of the original logits; rounding and clipping can change ordering. It is not the full method of Liang et al. because input perturbation is absent [20]. The temporary logit-exposing export used for the original maximum-logit and energy analyses was not retained. Those two scores are omitted from the revised primary comparisons rather than presented as reproducible new results.

### 2.10. Outcomes and Statistical Analysis

The primary outcomes have different denominators. The false-positive image rate is the proportion of NEG-SKIN images with at least one accepted detection. Candidate removal is the fraction of the 345 negative detection boxes rejected. Conditional retention is the fraction of already detected, reference-matched true-positive candidates retained. Overall lesion recall uses all annotated lesions, including those never detected, as the denominator. One-to-one greedy matching uses descending detection confidence and IoU ≥ 0.5. Image, source-photograph, candidate, and lesion results are identified explicitly; no patient-level endpoint can be estimated. Area under the receiver operating characteristic curve (AUROC), false-positive rate at 95% true-positive rate (FPR@95%TPR), margin ablation, and threshold sweeps are exploratory.

For major endpoints, 95% confidence intervals (CIs) use 10,000 percentile bootstrap resamples with seed 20260929. Resampling groups are the finest recoverable source families: 347 for NEG-SKIN, 93 for corrected POS-PI-GS, 111 for historical POS-PI-G, and 328 image identifiers for WOUND-NPI. Every group retains all associated copies and candidates, and paired before/after values are resampled together. These groups are not assumed to be distinct patients. Unknown repeated-patient dependence may narrow the intervals. Sparse stage-specific candidate proportions also have descriptive Wilson intervals [35], which do not adjust for clustering. The one-way removal architecture makes paired effect sizes more informative than a significance test based on discordant outcomes [36].

Intervals quantify sampling uncertainty within the observed pools. They do not include uncertainty from model training, threshold selection, incomplete overlap detection, label error, or unknown patient clustering. A bootstrap upper bound of 100% for retention does not demonstrate that rejection is harmless.

Finally, because the 1138 NEG-SKIN images were derived from 347 source photographs, we used two analyses to assess whether source photographs with more copies disproportionately influenced the false-positive image rate. First, for each source photograph, we calculated the proportion of its copies with a false positive and then averaged these proportions equally across the 347 sources. Second, in a conservative source-level analysis, a source photograph was considered to be false-positive if any of its copies produced a false positive.

### 2.11. Source Grouped Supervised Comparisons

We conducted a new internal gate-only experiment using the available accept/reject crop inventory. Negative groups use source-image content hashes; public positive groups remove augmentation suffixes; institutional positive groups remove repeated annotation-export prefixes and crop indices. Groups were assigned once to approximately 60% training, 20% calibration, and 20% test, using seed 20260929 and source-category stratification. Public augmented positive groups in calibration and test contribute one deterministic representative; all crops from a group stay in the same partition. There were 5096 training crops from 1534 groups, 1043 calibration crops from 511 groups, and 980 test crops from 512 groups. Group intersections were zero. These are recoverable source identifiers, not patient identifiers.

Three supervised binary models used exactly these partitions: YOLO26s-cls, ResNet18 [37], and MobileNetV3-small [38]. Initialization used the local pretrained 1000-class YOLO26s classifier or torchvision ImageNet-1K V1 weights, with a new two-class output head and every parameter trainable. Each model used 10 fixed epochs, AdamW, a learning rate of 0.0001, a weight decay of 0.001, a batch size of 64, and cross-entropy loss. The final epoch was evaluated without test-driven or calibration-driven checkpoint selection. Inputs are resized so that the shorter side is 224, then center-cropped to 224; training adds a horizontal flip probability of 0.5, a vertical flip of 0.3, and color jitter (brightness/contrast/saturation 0.2; hue 0.02). YOLO uses division by 255; torchvision models additionally use ImageNet mean and standard deviation. Training used PyTorch 2.6.0 and an NVIDIA RTX 3090 Ti.

For each model, calibration positive scores alone set the highest threshold, retaining at least 95%, 98%, or 99% of 305 positive calibration crops, with acceptance defined by score ≥ threshold. The threshold is then fixed for 273 positive and 707 negative test crops. The 98% target is a descriptive common comparison, not a clinically endorsed safety target; all three targets are retained in the result files. No test data select epochs or hyperparameters. This is a single train/calibration/test experiment, not nested cross-validation or an external clinical cohort. Crops remain related to the original upstream detector development and retain label-source confounding.

For additional challenge replay, the new classifiers receive crops from the unchanged historical detector boxes, whose saved coordinates are rounded to 0.1 pixels. RGB crops expand the box by a total 15% margin, then use the same resize, center crop, normalization, mixed-precision forward pass, and float32 softmax as the calibration/test analysis. Each model keeps its calibration-selected 98%-target threshold. No challenge scores select a model or threshold. The original gate-development source-family exclusion is retained for this comparison, but the positive images still overlap detector development.

### 2.12. Exploratory Error Coding

All 61 residual NEG-SKIN candidates at a detector confidence of 0.20 and gate threshold of 0.70 were examined using their source image and context crop. An AI-assisted descriptive visual review assigned one dominant appearance category per candidate, with rules and IDs stored in the annotation manifest. Categories concern visible regions rather than inferred network features or clinical diagnoses. The review was not blinded clinician adjudication and has no interrater reliability estimate. Both historical false-negative pressure ulcer cases were retained in the visual review, including the case removed from the corrected primary subset by the source-family rule.

### 2.13. Paired Detector Fine Tuning

Two additional detector runs started from the same retained original study checkpoint. The control reused 1340 positive training images; the other arm added 533 mined negative source images from the new training partition, with empty detection labels. No added negative images had identical file bytes to the 1466 negative challenge images. Both arms used 10 fixed epochs, AdamW, a learning rate of 0.0001, a weight decay of 0.001, a batch size of 16, 640-pixel input, a seed of 20260929, mixed precision, and mosaic/mixup disabled. Final-epoch weights were evaluated without challenge-based selection. Equal epochs mean unequal update counts because the augmented arm contains more images. The inherited 194-image validation split was not a new independent clinical cohort.

All three detector states, including the unmodified starting checkpoint, were evaluated with the same native PyTorch/Ultralytics runner, forced square 640 preprocessing, confidence at 0.20, class-agnostic IoU 0.6 duplicate removal, and one-to-one reference matching at IoU 0.5. No gate was applied. Recomputing the starting detector avoids comparing backend-dependent counts directly with historical ONNX outputs. Positive challenge images were previously seen during development of the starting checkpoint, so this is a controlled retrospective sensitivity experiment rather than independent detector validation.

## 3. Results

### 3.1. False-Positive Failure of the Closed-Set Architecture

On 1138 NEG-SKIN images, the original pipeline produced 345 candidate-level false positives across 269 images (23.6%; source-cluster 95% CI, 20.1–27.1%). These are outputs counted against the public Normal labels, not a prospective estimate of clinically adjudicated false-positive incidence.

The source-equal-weighted false-positive image rate was 25.0%, while 144/347 source photographs (41.5%) produced at least one false-positive copy. These complementary measures show the role of counting images versus source photographs. Differences across the supplied augmented copies indicate sensitivity to image transformations; they do not establish instability under new camera views or new clinical acquisitions.

**Figure 2:**
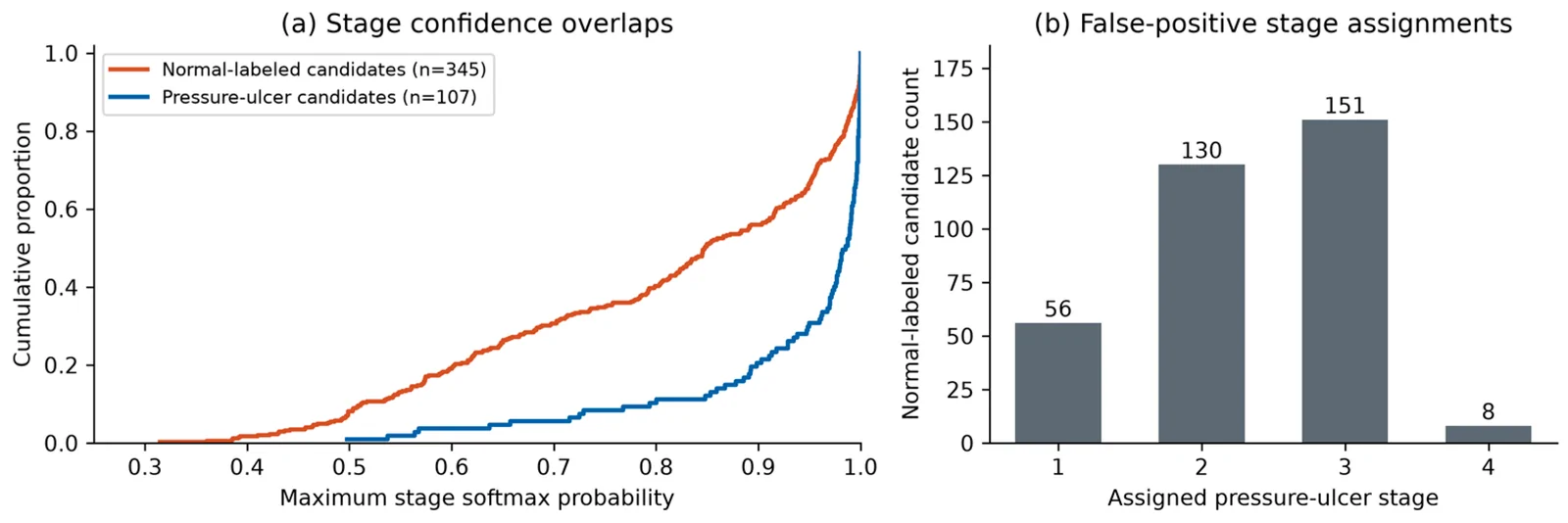
Stage confidence on Normal-labeled skin candidates. (**a**) Maximum softmax probability for 345 NEG-SKIN candidates and 107 matched candidates from the corrected POS-PI-GS subset. The positives remain associated with detector development. (**b**) Stage assignments for the negative candidates. Confidence among four stage labels does not establish lesion presence.

False-positive stage assignments were often confident (Figure 2). The mean MSP across 345 skin candidates was 0.804; a total of 44.1% exceeded 0.90 and 16.2% exceeded 0.99. The predicted stages were Stage 1 for 56 candidates, Stage 2 for 130, Stage 3 for 151, and Stage 4 for 8. These are model outputs on negative images, not clinical stage assessments.

### 3.2. Limitations of Raising the Detection Threshold

Increasing detector confidence from 0.20 to 0.40 reduced negative skin images with an output from 269 to 141 (Table 5). In the complete 229-image positive development pool, detected reference lesions decreased from 280/311 to 254/311. These historical recall values use a different denominator from the 110-lesion corrected gate analysis and do not constitute independent detector validation.

### 3.3. Effect of the Verification Gate and Sensitivity Cost

At the existing threshold of 0.70, 54/1138 images retained an output (4.7%; 95% CI, 3.0–6.7%) and 61/345 negative candidates remained. Candidate removal was 82.3% (95% CI, 75.7–88.3%). The paired image-rate change was −18.9 percentage points (95% CI, −22.1 to −15.7). The effect is specific to this challenge set and the frozen detector (Table 6).

In POS-PI-GS, the gate retained 106/107 detected reference lesions (99.1%; source-cluster 95% CI, 97.0–100%). Three reference lesions were already missed by the detector. Recall therefore changed from 107/110 (97.3%; 95% CI, 93.9–100%) to 106/110 (96.4%; 95% CI, 92.5–99.1%); the paired change was −0.91 percentage points (95% CI, −2.91 to 0.00). These remain development-associated estimates. For transparency, the historical 112-image subset retained 129/131 detections and had recall of 129/136 after the gate. Its two additional misses are still reviewed below; the apparently higher corrected retention reflects the changed subset, not an improved model.

There were 215 skin images with a false-positive output removed and no images with a newly introduced output. The latter is guaranteed by the filter architecture. The clinically relevant question is how the reduction balances additional missed lesions, not whether the one-way reduction is statistically detectable.

With equal weight for each of the 347 source photographs, the false-positive rate decreased from 25.0% to 4.4%. Under an any-copy-positive definition, it decreased from 144/347 to 31/347. The benefit therefore remains when augmented photographs are grouped, although neither analysis resolves patient dependence or acquisition-source bias.

### 3.4. Comparison with Training-Free Alternatives

**Figure 3:**
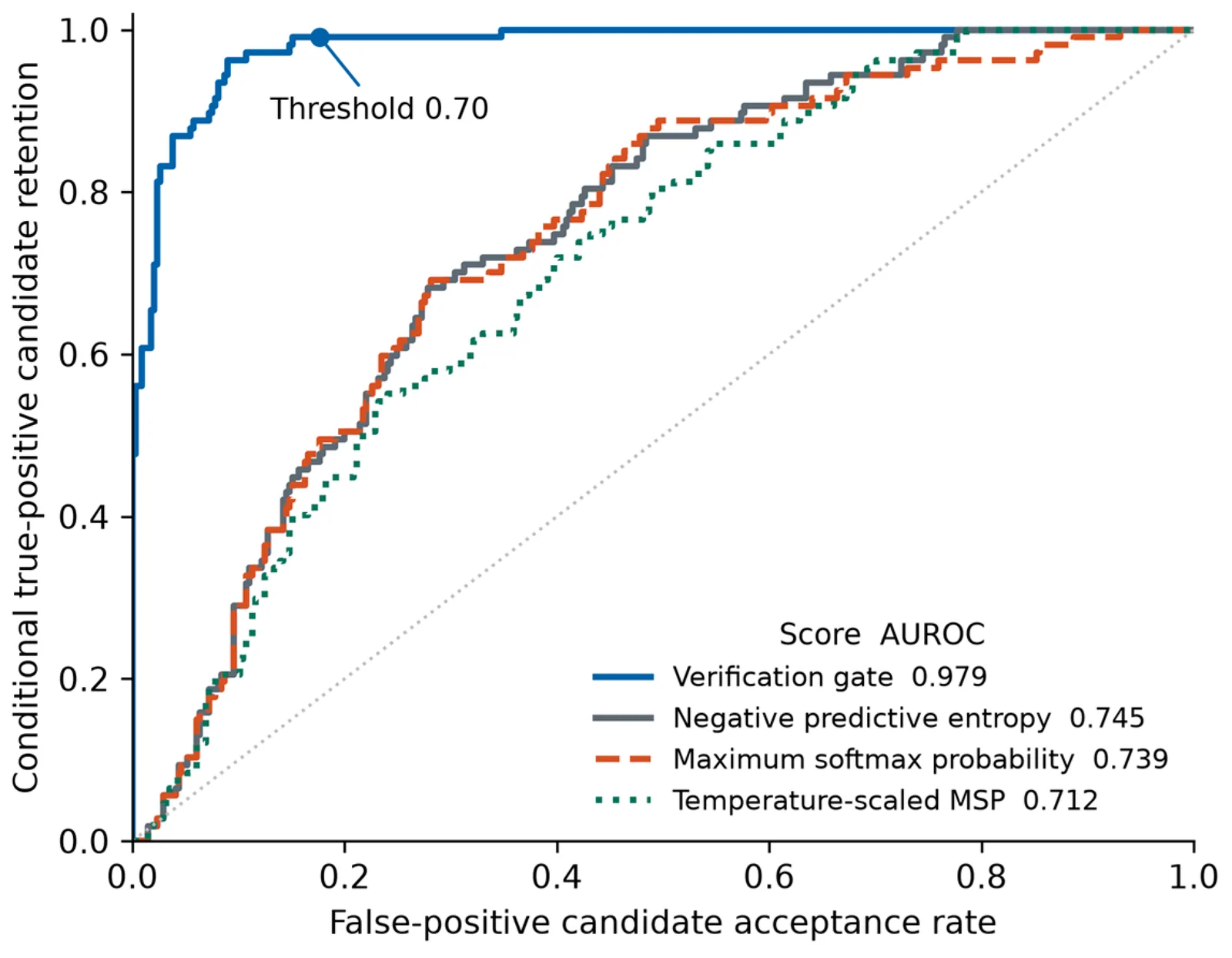
Receiver operating characteristic curves for candidate rejection. Curves use 107 matched POS-PI-GS candidates and 345 NEG-SKIN candidates. The positive subset is conservatively screened against gate-development source families but overlaps with detector training. Only reproducible scores are included. Complete-pool logit-based analyses appeared only in the original submission and are excluded from these revised comparisons.

On 107 POS-PI-GS matched candidates and 345 NEG-SKIN candidates, AUROC was 0.979 (0.964–0.990) for the gate, 0.745 (0.684–0.803) for NENT, 0.739 (0.677–0.799) for MSP, and 0.712 (0.636–0.790) for approximate temperature-scaled MSP (95% CIs; Figure 3). At an exploratory threshold retaining at least 95% of these positives, candidate-level false-positive rates were 9.0% for the gate and 69.3–73.0% for the confidence scores.

The gate-versus-confidence contrast depends on negative supervision and the chosen sources. It cannot establish a new algorithmic advance, and its AUROC is not a measure of clinical sensitivity or of wound-type diagnosis. The same caveat applies to the new source-grouped classifier comparison below.

Table 7 selects each method’s threshold on POS-PI-GS to meet the stated retention target, then describes negative image outputs. These are within-sample operating characteristics. They are distinguished from the source-separated calibration/test experiment, where the test positives play no role in choosing thresholds.

In the corrected subset, a required retention of 99% permits one rejection: 106/107 candidates remain at a gate threshold of 0.890639. The false-positive image rate is 4.2%. This differs from the original 112-image analysis because the source-family correction changes the low-score tail. The change must not be interpreted as a better model or a newly validated clinical operating point.

### 3.5. Rejected Lesions and Staging Behavior

The original 112-image subset contained two additional rejected true lesions: a Stage 1 lesion with detector confidence of 0.385 and gate score of 0.054, and a Stage 3 lesion with detector confidence of 0.796 and gate score of 0.0715. The Stage 1 image was subsequently excluded from POS-PI-GS because its source family appears in available gate-development material; the Stage 3 image remains. Both are displayed so that the provenance correction does not erase the original safety signal. The Stage 1 detected box occupied 44.9% of its image, which also contains visible pre-existing masking or image alteration. The scores do not establish why the model rejected either case.

Using the inherited per-lesion annotations, corrected subset retention was 11/11 for Stage 1, 58/58 for Stage 2, 27/28 for Stage 3, and 10/10 for Stage 4. Descriptive Wilson 95% CIs were 74.1–100%, 93.8–100%, 82.3–99.4%, and 72.2–100%, respectively. The revision corrects the original use of image-filename stage labels: some images contain lesions with different annotated stages. The historical 112-image subset has corresponding counts of 12/13, 76/76, 31/32, and 10/10. Its Stage 1 miss remains a safety concern; source-based exclusion and small perfect proportions do not demonstrate stage-specific safety.

The classifier receives identical inputs and weights for accepted candidates, so their stage predictions are unchanged by construction. Rejected candidates receive no prediction. This property is not evidence of unchanged system-level staging accuracy, which cannot be estimated independently with the available positive data.

### 3.6. Context-Margin Ablation

At context margins of 0%, 8%, 15%, and 25%, residual negative candidates numbered 78, 71, 61, and 58; corrected positive retention remained 106/107. AUROC ranged from 0.9772 to 0.9790. This inference-time ablation shows sensitivity to surrounding context, but cannot separate useful lesion surroundings from nuisance acquisition features. It neither proves morphological learning nor establishes 15% as optimal.

### 3.7. Failure-Mode Stress Test in Non-Pressure Wounds

WOUND-NPI contains 328 public diabetic foot ulcer images with 355 annotated wounds. It is an alternative wound dataset used to test a known limitation, not an external pressure ulcer validation cohort. Its etiologic labels were inherited from the public release.

**Figure 4:**
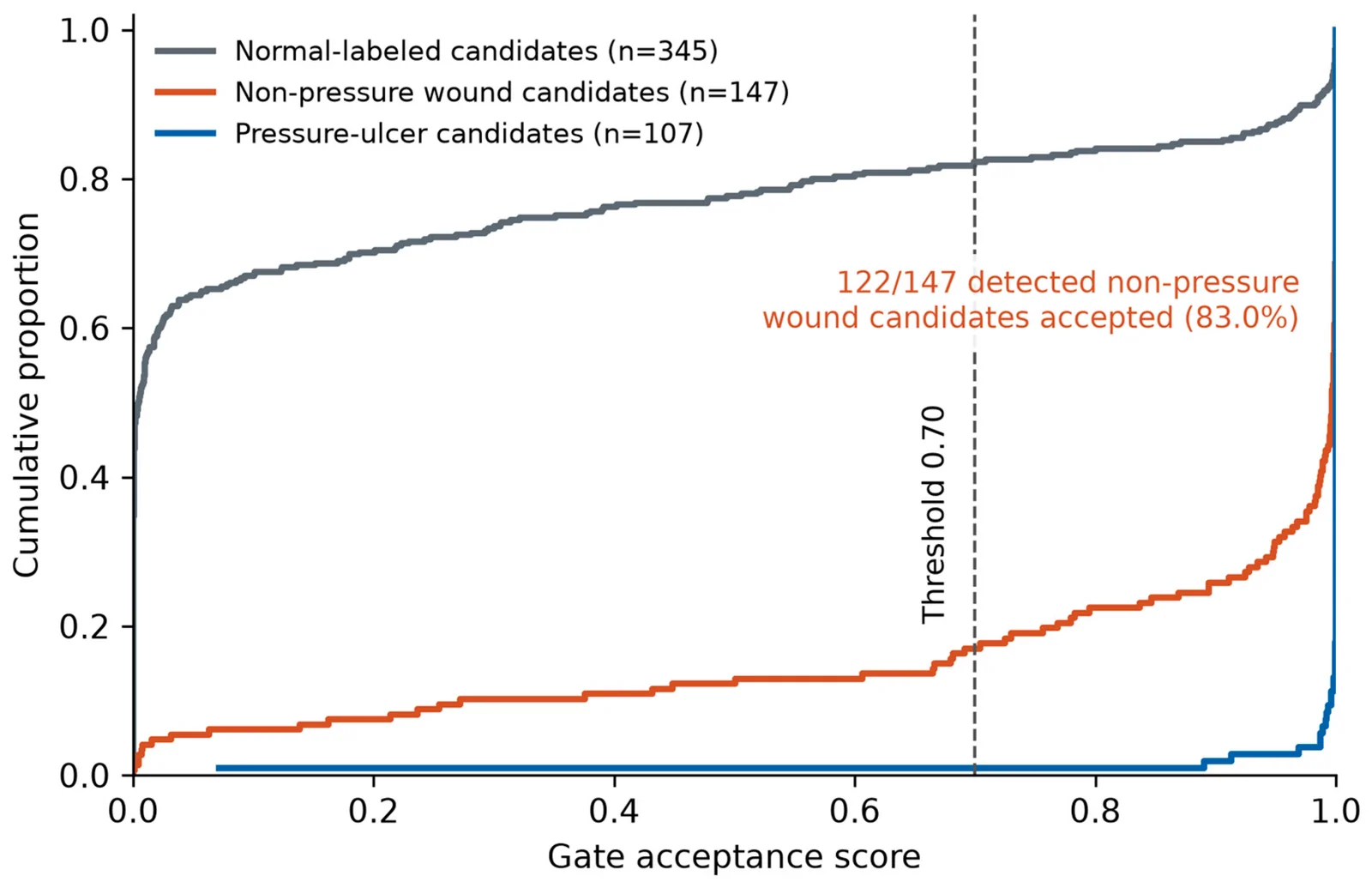
Distributions of gate acceptance scores by condition. Acceptance scores for 345 NEG-SKIN candidates, 147 WOUND-NPI matched candidates, and 107 corrected POS-PI-GS matched candidates. P(accept) is a binary training-task score, not a probability of pressure ulcer etiology. The operating threshold is 0.70.

Figure 4 shows the distributions of gate acceptance scores across the three conditions. The gate accepted 122/147 reference-matched non-pressure-wound candidates (83.0%; image-cluster 95% CI, 76.6–89.0%). Considering all candidates, 183/258 passed and 178/328 images emitted at least one pressure ulcer stage (54.3%; 95% CI, 49.1–59.8%). Matched-candidate acceptance and image-level output are different endpoints. Mean stage MSP was 0.905 for accepted matched candidates. Gate AUROC was 0.894 for other wounds versus normal-labeled skin and 0.814 for corrected POS-PI-GS versus other wounds (Table 8). These results demonstrate a substantial unresolved risk of assigning inappropriate pressure ulcer stages to other wound types.

The evaluated pipeline has no mechanism that reliably prevents staging a diabetic foot ulcer or another accepted non-pressure wound. Clinical pressure etiology would need to be confirmed before a stage is interpreted; this is a proposed use restriction, not a tested safeguard in the application. Developing wound-type differentiation requires appropriately labeled dermatitis, skin tears, vascular ulcers, surgical wounds, burns, and other mimics, together with an option to abstain when etiology is uncertain.

### 3.8. Descriptive Comparisons Across Sources and Stages

Table 8 combines the available sets to describe discrimination under different case and source mixtures. Acquisition context includes camera, lighting, field of view, body site, background, and preprocessing. Device metadata and matched institutional negatives were unavailable. These comparisons therefore probe sensitivity to the chosen comparison group; they do not control acquisition context.

Comparison C uses two different public datasets with broadly similar foot anatomy and the same released resolution. Public hosting does not imply the same institution, device, protocol, or patients. Its AUROC of 0.894 therefore cannot rule out source-related shortcuts. Likewise, the lower AUROC in B than A could reflect wound morphology, source differences, or both; these factors are not identifiable from this design. Stage-stratified values remain cross-source comparisons with particularly small Stage 1 and Stage 4 samples. A matched-device, matched-workflow positive/negative cohort is required to isolate acquisition confounding.

### 3.9. Deployment Overhead

Adding the gate increased model assets from 79.6 MB to 101.4 MB and the arm64-v8a deployment package from 94.7 MB to 107.3 MB (90.3 MiB and 102.3 MiB, respectively).

Latency was measured using a Pixel 8 API 35 emulator (x86_64, 4 vCPUs), the default ONNX Runtime CPU execution provider without a registered hardware delegate, four intra-operation threads (the smaller of the core count and four), and one detection candidate. After excluding session loading and the first inference as warm-up, repeated runs on a 601 × 564 input yielded inference times of 478–851 ms across four measurements. The first call, including loading of all three models, required 2.9 s.

### 3.10. Newly Trained Supervised Baselines

At thresholds selected to retain at least 98% of calibration positives, the three models retained 266, 267, and 269 of 273 test-positive crops and rejected 703, 699, and 698 of 707 test-negative crops (Table 9). The evidence therefore supports useful internal crop discrimination across the evaluated architectures, without a demonstrated YOLO-specific advantage. The new test crops and thresholds differ from the deployed-model challenge analysis, so these values must not be substituted for end-to-end recall or the historical 0.70 operating point.

All models retained 299/305 calibration positives at this target. Test confidence intervals cluster 254 positive and 258 negative source groups. For the 95% calibration target, observed test retention was 96.3% for YOLO26s, 93.8% for ResNet18, and 94.5% for MobileNetV3-small; a calibration target is therefore not a test-time guarantee. At the 98% target, institutional positive retention was 156/160, 156/160, and 157/160, respectively; public positive retention was 110/113, 111/113, and 112/113. These strata remain acquired through different pathways and do not constitute a source-matched experiment. The unusually high crop AUROCs should be read in that restricted setting.

At frozen calibration-selected thresholds, challenge replay yielded 47, 51, and 55 residual negative candidates for YOLO26s, ResNet18, and MobileNetV3-small. Affected NEG-SKIN images were 43/1138 (3.8%), 45/1138 (4.0%), and 48/1138 (4.2%); corrected positive retention was 106/107, 107/107, and 106/107. Source-cluster intervals are provided with the replay results. This extends the non-YOLO comparison to the challenge distribution without removing detector-development dependence. The same gates accepted 102/147, 133/147, and 114/147 non-pressure-wound candidates, preserving the etiology limitation.

### 3.11. Residual Candidate Appearance Categories

The 61 residual candidates occurred in 54 released images from 31 source families. Their appearance was more varied than the original description of narrow red glossy regions (Table 10). Nail or nail-fold regions accounted for 26/61 candidates; broad foot or multiple-toe regions, creases and boundaries, textured skin, focal heterogeneous patches, and embedded graphics made up the remainder. Counts describe candidate appearances and include correlated augmentation copies. They do not identify the features used by the network.

**Figure 5:**
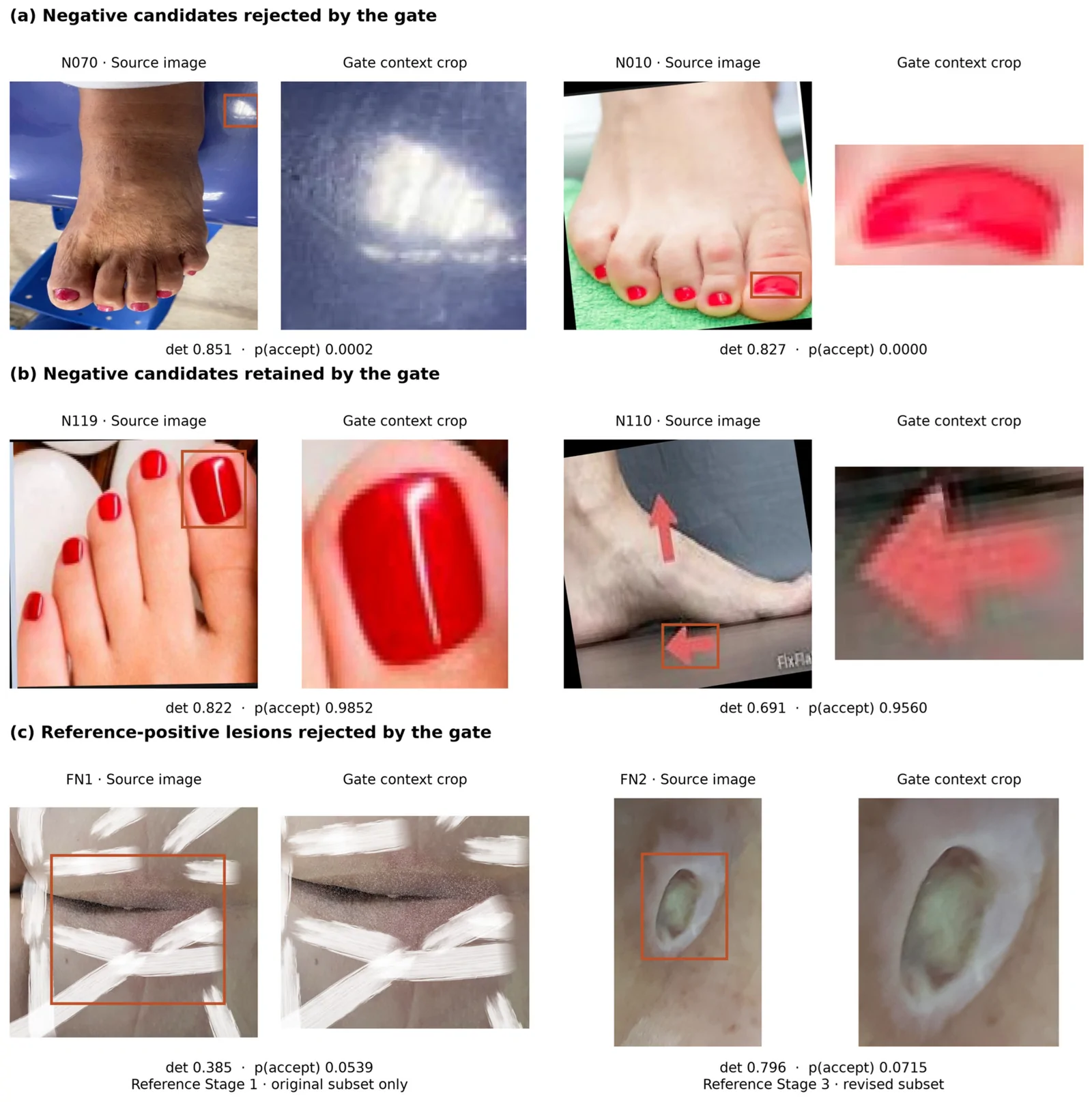
Candidate examples and the two additional true lesions rejected by the gate. Panels show rejected negative candidates, retained negative candidates from different source groups, and both historical gate-rejected true lesions. Boxes mark the detector region, crops retain its 15% context margin, and P(accept) is the gate score. Reference stages are inherited labels, not new adjudications. FN1 is excluded from the corrected primary subset by the source-family rule; FN2 remains. Pre-existing image markings or masks are preserved. IDs link examples to the figure-selection and taxonomy manifests. Display enlargement adds no image detail.

One category is assigned per candidate. Source-family counts across categories are not additive. Focal abnormal-looking patches remain etiologically unverified under the original Normal labels; their presence limits the clinical certainty of the negative reference standard. The two false-negative examples in Figure 5 remain separate from this negative-candidate taxonomy. The complete coding manifest permits author review and future clinician adjudication.

### 3.12. Direct Detector Retraining with Negative Images

Under the common native PyTorch runner, the original detector produced 344 negative candidates on 268/1138 images, one candidate/image fewer than the historical ONNX reproduction. Positive-only fine-tuning produced 411 candidates on 321 images. Adding the mined negative full images reduced these to 81 candidates on 62 images, while matched lesions changed from 107/110 to 106/110 (Table 11). Relative to the recomputed original detector, candidate reduction was 76.5% (95% CI, 68.8–83.9%) and the negative image-rate change was −18.1 percentage points (95% CI, −21.4 to −14.9).

The net loss of one detected lesion conceals three lost and two newly detected reference lesions relative to the original detector. The paired recall change was −0.91 percentage points (95% CI, −4.85 to 2.86), so it must not be described as the same false-negative pattern as the gate. Negative-image training is a viable alternative in this experiment; the results do not establish superiority, noninferiority, or equivalence of either approach. The short fixed schedule, unequal update counts, backend differences from the historical gate analysis, and prior positive exposure restrict interpretation.

## 4. Discussion

### 4.1. Confidence and Candidate Acceptance

The results illustrate a limitation of using stage confidence as a surrogate for lesion presence: a classifier can prefer one stage strongly when all available labels are inappropriate. This is consistent with open-set recognition concerns [17,19,23]. Hard-negative supervision supplies information about detector errors that the original stage classifier did not receive. However, a high gate score has the same interpretive boundary: it expresses agreement with its accept training class, and cannot certify wound etiology or clinical safety.

### 4.2. False Negatives and Clinical Acceptability

The two historical gate-rejected true lesions represent a real cost. There was no clinical noninferiority margin, patient-outcome analysis, expert utility assessment, or independent safety cohort. We cannot judge that loss clinically acceptable. The Stage 1 case remains relevant even after provenance-based exclusion from the corrected subset, while the retained Stage 3 case shows that rejection is not restricted to early injury. Both scores were below 0.08, so a narrow review band around 0.70 would not have recovered them. The evaluated application silently suppresses rejected candidates and has no recovery mechanism.

### 4.3. Role of the Proposed Module and Interpretation of Metrics

The candidate verification gate is a standard supervised binary classifier placed between two frozen modules. Its contribution is modular integration and a focused evaluation of detector-derived false-positive candidates, rather than technical novelty in classification. The architecture permits a separate rejection threshold and preserves the stage computation for accepted candidates, but adds inference cost and an additional opportunity to miss a lesion. Whether this tradeoff is preferable depends on comparison with other supervised uses of the same negative data.

A proposed recovery pathway would preserve rejected boxes and permit clinician inspection, recapture, or an explicit uncertain result instead of presenting rejection as absence of a lesion. Patients with clinical suspicion should remain assessable regardless of model output, including when the detector proposes no box. Such a pathway could alter review workload and sensitivity; it was not implemented or evaluated in this study and requires a prospective workflow study before a safety benefit can be claimed.

Negative challenge-set suppression is the best supported result because it is measured on images with no identified development overlap. Positive retention is less secure: the stronger family audit removes additional related images, but every remaining pressure ulcer image was still used in detector development. Source grouping improves the accounting; it cannot undo development reuse, recover missing patient identity, or demonstrate performance in a new institution.

### 4.4. Scope of Comparison

The new ResNet18 and MobileNetV3-small models demonstrate that the observed internal crop-level discrimination is not unique to a YOLO classifier. They receive the same negative supervision and source partitions as the new YOLO gate. Similar estimates and overlapping intervals do not prove equivalence; one seed, a fixed short training schedule, different pretrained representations, and residual source confounding limit architectural conclusions. A five-class staging model and explicit outlier-exposure objective were not trained. Their possible advantages remain open rather than being inferred from the training-free comparisons. Changes to the detector, acquisition device, or imaging workflow can alter the candidate distribution and require renewed calibration and evaluation before use.

The paired detector experiment also supports background retraining as a viable alternative. Its similar aggregate recall does not imply the same missed-lesion pattern, and different execution backends and training update counts preclude a superiority or equivalence claim against the historical gate. The relevant implementation decision must consider missed cases, review workflow, computational cost, and generalization in an independent cohort.

### 4.5. Limitations

The principal limitation is the absence of an independent positive patient cohort. All positive evaluation images participated in detector development, and patient identifiers were unavailable. Distinct images may depict the same patient or wound episode, so source-image grouping is not patient separation. Gate retention, discrimination, and recall can remain optimistic through both correlated photographs and detector-derived crop selection. Patient-level CIs cannot be recovered from the available files. The study also does not estimate independent Stage 1–4 accuracy or clinical outcomes.

Acquisition source is confounded with label. Clinical positives and public negatives differ in provenance, and even the two public foot datasets are not camera- or institution-matched. Consequently, the evidence does not determine whether morphology or nuisance context dominates the gate decision. Color augmentation and crop-margin ablation are not substitutes for an acquisition-matched experiment.

The skin challenge set is dominated by foot images and augmented copies of a limited number of source photographs. Sacral and coccygeal skin, heels under matched clinical conditions, postoperative wounds, dermatitis, skin tears, and burns were not systematically sampled or annotated. The available data cannot support site-specific negative performance for these categories. Other-wound rejection is also inadequate, and unstageable injury and DTPI remain outside the four-stage output space.

Additional development analyses use the available crops and source identifiers, whose lineage is imperfect. They do not replace an external positive cohort. The retrospective visual error taxonomy is descriptive, without blinded clinician adjudication or interrater agreement. Historical model-validation accuracy and threshold choice should be read within these provenance and selection limitations.

### 4.6. Future Work

A confirmatory study should acquire pressure ulcers and negative or mimicking findings within the same sites, devices, and workflows, assign patient and wound-episode identifiers before splitting, and reserve an untouched external cohort. Model fitting, hyperparameter selection, threshold calibration, and evaluation should use separate groups. The protocol should define an acceptable recall loss with clinicians, measure the lower confidence bound rather than only a point estimate, and record missed-lesion severity, review workload, skin tone, anatomical site, and recovery outcomes. Neither a fresh patient cohort nor these clinical outcomes can be created by re-splitting the current images.

## 5. Conclusions

Negative supervision reduced false-positive outputs through both candidate verification and direct detector fine-tuning in these retrospective experiments. A separate gate is therefore a modular implementation option, not a demonstrated uniquely necessary or superior solution. The source-family and per-lesion-label corrections also show why data provenance must accompany aggregate performance claims.

The present evidence does not justify autonomous exclusion of pressure injury, clinical acceptability of the missed lesions, or staging of other wound types. The next decisive test is a prospectively specified, patient-separated evaluation with matched acquisition conditions and a review pathway for uncertain or rejected findings.

## Figures and Tables

**Table 1:** Data roles for the deployed components and the retrospective evaluation. Training and validation counts refer to complete images for the detector and crops for the classifiers. Development-validation values describe model development; they are not independent pipeline performance. The positive challenge images overlap detector and stage-classifier development. Overlap counts are reported separately. Here, mAP50 denotes mean average precision at an IoU threshold of 0.50.

Component and Initialization	Training	Development Validation	Threshold Calibration	Challenge-Set Role
Detector YOLO26s pretrained checkpoint	1340 images	194 images; historical mAP50 0.650	No gate threshold fitted here	All 229 positive files reused from detector development
Verification gate YOLO26s-cls pretrained checkpoint	7541 crops 4262 accept; 3279 reject	838 crops 375 accept; 463 reject	255 negative images; 199 matched positive candidates	1466 negatives; corrected 94 positive files remain detector-associated
Stage classifier YOLO26m-cls pretrained checkpoint	13,304 crops	375 crops; historical top-1 0.808	None for the stage output	All positive reference lesions overlap development; no independent staging accuracy
Revision-only binary classifiers: three pretrained architectures	5096 crops; 1534 source groups	No validation-based model selection; fixed epoch 10	1043 crops; 511 groups	980 crops; 512 source groups; internal gate-only test

The originally reported 0.988 gate development accuracy is not used as confirmatory evidence. The available saved gate data contain source-disjoint reject partitions, contrary to the original crop-split description; historical checkpoint membership is not fully recoverable from the current directory alone. The newly controlled experiment is reported separately in Section 2.11. The stage-classifier training crops include 11,346 public staging crops and 1958 institutional lesion-box crops; validation includes 92 and 283, respectively.

**Table 2:** Training settings for the three models. This Table records the historical training configuration. Saved run arguments specify pretrained checkpoint initialization for all three components; each was then fine-tuned on task-specific data. The detector used yolo26s.pt, the gate used yolo26s-cls.pt, and the stage classifier used yolo26m-cls.pt. These were not random-initialization experiments. The exact pretraining data mixture is not encoded in the retained run arguments. Optimizer auto-selection was used; lr0 is a configured value rather than an assurance of the resolved optimizer learning rate. Detection-specific settings such as mosaic, multi_scale, and copy_paste should not be interpreted as active classification augmentations merely because they appear in saved configuration files.

Item	Detector	Verification Gate	Stage Classifier
Input size/batch	640/16	224/64	224/64
Epochs (patience/run)	200 (50/178)	80 (20/60)	150 (20/56)
Optimizer/lr0	AdamW/0.01	AdamW/0.01	SGD/0.01
weight decay/dropout	0.0005/0.0	0.001/0.3	0.001/0.3
hsv_h/hsv_s/hsv_v	0.0/0.3/0.3	0.02/0.6/0.4	0.015/0.7/0.4
degrees/translate/scale	0.0/0.1/0.5	15.0/0.1/0.5	0.0/0.1/0.5
fliplr/flipud/erasing	0.5/0.0/0.4	0.5/0.3/0.4	0.5/0.0/0.4
mosaic/multi_scale/copy_paste	1.0/0.5/0.3	1.0/0.0/0.0	1.0/0.0/0.0
Total training time	2932 s	351 s	1086 s

**Table 3:** Composition of the evaluation data. Parentheses give recovered source-family counts, not patient counts. Matched candidates use detection confidence 0.20 and IoU ≥ 0.5. NEG-SKIN includes every candidate. WOUND-NPI has 258 total candidates, POS-PI 331, historical POS-PI-G 147, and corrected POS-PI-GS 114; only reference-matched candidates enter lesion retention. The 229 POS-PI files collapse to 226 normalized source families.

Set	Composition	Source	Images (Source Families)	Reference Lesions	Matched Candidates
NEG-SKIN	Normal-labeled skin	Public Ulcer Classification v2	1138 (347)	0	345 (all candidates)
WOUND-NPI	Other wounds	Public Foot Ulcer v2	328 (328)	355	147
POS-PI	Complete pressure ulcer pool	Institutional development data	229 (226)	311	280
POS-PI-G	Historical exact-file exclusion	Subset of POS-PI	112 (111)	136	131
POS-PI-GS	Corrected source-family exclusion	Subset of POS-PI-G	94 (93)	110	107

**Table 4:** Audit of overlap with training and threshold-calibration data. Counts are image files unless stated otherwise. Historical POS-PI gate membership comprised 85 training and 32 validation images by exact identity. Eighteen additional files in the historical 112-image subset are flagged by normalized source-family relations to available gate-development material. The corrected 94-image subset excludes these conservatively. Zero identified overlap means no match under the stated audit, not certified patient independence. Gate development includes both fitting and model-selection data.

Set	Files	Detector Train/Val	Gate Development by Exact ID	Additional Family Flags	Stage Development	Gate Calibration by Exact ID
NEG-SKIN	1138	0/0	0	0 identified	0	0
WOUND-NPI	328	0/0	0	0 identified	0	0
POS-PI	229	197/32	117	18 among POS-PI-G	229	32
POS-PI-G	112	112/0	0	18	112	0
POS-PI-GS	94	94/0	0	0 after exclusions	94	0

**Table 5:** Effect of increasing the detection-confidence threshold. This historical detector-threshold analysis uses 1138 negative images and all 229 POS-PI development-associated images with 311 lesions. Its negative confidence intervals retain the original source-cluster bootstrap estimates, unlike the 10,000-resample revision analyses. The positive denominator differs from that of the corrected gate analysis; these are not independent patient sensitivity estimates.

Detection Threshold	False-Positive Images	False-Positive Image Rate (95% CI)	False-Positive Detections	Detected Lesions	Overall Lesion Recall
0.10	404	35.5% (31.7–39.4)	542	288/311	92.6%
0.20 (deployment default)	269	23.6% (20.2–27.2)	345	280/311	90.0%
0.30	192	16.9% (14.0–19.9)	248	267/311	85.9%
0.40	141	12.4% (9.9–15.0)	175	254/311	81.7%

**Table 6:** Gate-threshold sweep. Negative outcomes use 1138 images from 347 source photographs. Positive outcomes use the corrected POS-PI-GS subset: 94 images, 93 source families, 110 lesions, and 107 detected true-positive candidates. Detection confidence is 0.20. Confidence intervals resample source families. Thresholds other than 0.70 are exploratory.

Gate Threshold	Remaining Negative Candidates	Candidate Removal (95% CI)	Negative Image Rate (95% CI)	Positive Retention	Lesion Recall (95% CI)
No gate	345	0.0% (0.0–0.0)	269/1138; 23.6% (20.1–27.1)	107/107; 100.0%	107/110; 97.3% (93.9–100.0)
0.3	92	73.3% (65.1–81.2)	75/1138; 6.6% (4.6–8.9)	106/107; 99.1%	106/110; 96.4% (92.5–99.1)
0.5	77	77.7% (70.6–84.4)	66/1138; 5.8% (3.9–7.8)	106/107; 99.1%	106/110; 96.4% (92.5–99.1)
0.70 existing	61	82.3% (75.7–88.3)	54/1138; 4.7% (3.0–6.7)	106/107; 99.1%	106/110; 96.4% (92.5–99.1)
0.9	52	84.9% (78.8–90.4)	48/1138; 4.2% (2.6–6.1)	105/107; 98.1%	105/110; 95.5% (91.3–99.1)
0.95	44	87.2% (81.8–92.2)	42/1138; 3.7% (2.2–5.4)	104/107; 97.2%	104/110; 94.5% (90.1–98.2)

**Table 7:** Post hoc matched-tradeoff analysis at fixed conditional retention requirements. Each cell reports the negative image rate and achieved positive retention where applicable. Thresholds are chosen using the same 107 positive candidates later used to describe achieved retention. All methods achieved 102/107, 104/107, 105/107, and 106/107 at targets 95%, 97%, 98%, and 99%, respectively. Approximate temperature scaling is reconstructed from rounded probabilities; no independent threshold calibration is implied.

Required Retention	Achieved Retention	Gate Threshold	Gate Image Rate	NENT Image Rate	MSP Image Rate	Approx. TS Image Rate
95.0%	102/107	0.987197	2.6%	16.5%	17.2%	15.3%
97.0%	104/107	0.968945	3.2%	17.0%	20.0%	16.6%
98.0%	105/107	0.912825	4.2%	17.4%	20.0%	17.7%
99.0%	106/107	0.890639	4.2%	17.5%	20.9%	17.7%

**Table 8:** Discrimination by the gate acceptance score. A compares corrected POS-PI-GS with NEG-SKIN; B compares the same positives with WOUND-NPI; C compares WOUND-NPI with NEG-SKIN. Stage rows stratify A. Confidence intervals use 10,000 resamples of source families or image identifiers. C compares separate public datasets and is not matched for camera, institution, or acquisition protocol.

Comparison	Positive Candidates	Negative Candidates	Acquisition Relation	AUROC (95% CI)
A	POS-PI-GS (107)	NEG-SKIN (345)	Institutional versus public	0.979 (0.964–0.990)
B	POS-PI-GS (107)	WOUND-NPI (147)	Institutional versus public	0.814 (0.759–0.863)
C	WOUND-NPI (147)	NEG-SKIN (345)	Separate public foot datasets; device/site unknown	0.894 (0.852–0.931)
A-1	Stage 1 (11)	NEG-SKIN (345)	Institutional versus public	0.993 (0.982–1.000)
A-2	Stage 2 (58)	NEG-SKIN (345)	Institutional versus public	0.980 (0.965–0.991)
A-3	Stage 3 (28)	NEG-SKIN (345)	Institutional versus public	0.969 (0.937–0.991)
A-4	Stage 4 (10)	NEG-SKIN (345)	Institutional versus public	0.985 (0.966–0.996)

**Table 9:** New source-grouped gate-only test at separately calibrated 98% retention targets.

Model	Calibrated Threshold	Positive Retention (95% CI)	Negative Rejection (95% CI)	AUROC (95% CI)
YOLO26s-cls	0.44971	266/273; 97.4% (95.3–99.3)	703/707; 99.4% (98.6–100.0)	0.999 (0.998–1.000)
ResNet18	0.77138	267/273; 97.8% (95.9–99.3)	699/707; 98.9% (97.4–99.9)	0.999 (0.997–1.000)
MobileNetV3-small	0.60915	269/273; 98.5% (96.7–100.0)	698/707; 98.7% (97.5–99.6)	0.999 (0.998–1.000)

**Table 10:** Exploratory visual taxonomy of residual negative candidates.

Dominant Visible Region	Candidates	Percent	Source Families
Nail or nail-fold region	26	42.6%	11
Broad foot or multiple-toe region	11	18.0%	7
Skin crease or skin-background boundary	9	14.8%	8
Dry or yellow textured skin surface	5	8.2%	3
Focal heterogeneous skin patch	6	9.8%	2
Embedded graphic or background object	4	6.6%	2

**Table 11:** Paired detector fine-tuning comparison without a verification gate.

Detector State	Negative Candidates	Negative Image Output (95% CI)	Detected Lesions	Lesion Recall (95% CI)
Original checkpoint	344	268/1138; 23.6% (20.0–27.1)	107/110	97.3% (93.9–100.0)
Positive-only fine-tuning	411	321/1138; 28.2% (24.5–32.0)	107/110	97.3% (94.0–100.0)
Positive plus mined negatives	81	62/1138; 5.4% (3.7–7.3)	106/110	96.4% (92.6–99.1)

## Data Availability

The public source releases are Open Images V7 [33], PU_CLASIFFIER v1 (https://universe.roboflow.com/stage2-n7xya/pu_clasiffier (accessed on 12 August 2026)), Ulcer Classification v2 (https://universe.roboflow.com/lookinside/ulcer-classification/dataset/2 (accessed on 12 August 2026)), and Foot Ulcer v2 (https://universe.roboflow.com/medica/foot-ulcer-10nf1/dataset/2 (accessed on 12 August 2026)). Their source images and annotations are subject to the original licenses. Institutional images and annotations are not publicly deposited because they contain clinical photographs. The revision materials include analysis scripts, split manifests, derived evaluation tables, and a file inventory with hashes for the locally available model and result artifacts. These materials are prepared for author review; no public repository or permanent archive accession has yet been established. Original model weights, training logs, and permitted de-identified evaluation records can be requested from the corresponding author subject to institutional and licensing review. The temporary logit-exposing export used for the original energy and maximum-logit analyses was not retained. Publication of a reproducibility package and any clinical-image access remain subject to the corresponding author’s authorization; no unavailable component is described as publicly accessible.

## Abbreviations

The following abbreviations are used in this manuscript:

AUROC	Area under the receiver operating characteristic curve
CI	Confidence interval
CLAIM	Checklist for Artificial Intelligence in Medical Imaging
DTPI	Deep tissue pressure injury
EPUAP	European Pressure Ulcer Advisory Panel
FPR@95%TPR	False-positive rate at 95% true-positive rate
IoU	Intersection over union
mAP	Mean average precision
MSP	Maximum softmax probability
NPIAP	National Pressure Injury Advisory Panel
ONNX	Open Neural Network Exchange
TS	Temperature scaling
